# Laser Optofluidics in Fighting Multiple Drug Resistance

**DOI:** 10.2174/1874285801812010008

**Published:** 2018-02-28

**Authors:** Thodoris D. Karapantsios

**Affiliations:** Aristotle University of Thessaloniki, Greece

The book combines fields which experience increasing interest in recent years: optofluidics, microfluidics and fighting multiple drug resistance (MDR) seen as part of infectious diseases domain. Ther eported results are of interest for life sciences, environment quality control and biomedical basic and applied research. Biomedical specialists, chemists, physicists, public health experts and even outer space researchers may be among target readers, as well as students engaged in these fields.

The book shows a convincing and useful connection between optofluidics and MDR studies. It is distributed along 18 chapters written by 40 authors from 9 countries and 12 laboratories.

A set of chapters informs readers about selected non-antibiotic medicines which are exposed to UV pulsed laser beams for generating photoproducts with enhanced properties in fighting MDR. Such parent compounds are phenothiazines, quinazolines and hydantoin derivatives which do not have normally significant effects on bacteria or tumour tissues, but after being exposed to laser radiation in water (chosen as a biocompatible liquid) solutions, generate photoproducts with individual or synergistic effects on biological targets.

The book shows the most recent results in the action of exposed chlorpromazine and thioridazine on Gram-positive and Gram-negative bacteria and their enhanced antibacterial and antibiofilm activity.

Complementary data about the effects of two cytostatics, methotrexate and 5-Fluorouracil, exposed to optical radiation on eye pseudotumours are synthesised; showing that mixtures of photoproducts generated from them have higher anti-inflammatory effects then their parent compounds.

These data are correlated with reports about microfluidic properties for microdroplets serving as vehicles for the transport of medicines to targets.

